# A Three-Month Clinical Trial on the Efficacy of Hyaluronic Acid Adjunctive Non-Surgical Therapy for Periodontitis in Patients with Type 2 Diabetes Mellitus

**DOI:** 10.3390/biomedicines12112516

**Published:** 2024-11-04

**Authors:** Iwona Olszewska-Czyz, Ewa Michalak, Agata Dudzik

**Affiliations:** Department of Prophylaxis, Periodontology and Oral Pathology, Dental Institute, Medical Faculty, Jagielonian University, 30002 Krakow, Poland; ewa3.michalak@uj.edu.pl (E.M.); agata.skrzypek@uj.edu.pl (A.D.)

**Keywords:** hyaluronic acid, non-surgical periodontal treatment, periodontitis, diabetes

## Abstract

**Background/Objectives**: Conventional periodontal treatment for patients with diabetes has shown promising results, primarily focusing on glycated hemoglobin (HbA1c) levels as an endpoint measure. The properties of hyaluronic acid (HA) have been harnessed in various periodontal therapies, and it is a promising agent also in a non-surgical approach. The aim of this clinical trial was to assess the efficacy of hyaluronic acid in a local adjunctive non-surgical treatment for periodontitis in patients with type 2 diabetes. **Methods**: Eighty adult participants with well-controlled type 2 diabetes (HbA1c 7% (53 mmol/mol) or less) took part in the trial. The clinical parameters of periodontitis as well as the glycated hemoglobin (HbA1c) levels were evaluated, and an analysis of the potential differences between the control (placebo) and intervention (HA) groups was performed. **Results/Conclusions**: A decrease in all the clinical values of periodontitis after treatment was observed in the vast majority of patients in both groups. Differences in the clinical parameters were observed 12 weeks after the intervention between the patients in the placebo and HA therapy groups. Bleeding on probing (BoP) was reduced in the control group to 15–25% and was approximately 5.5% more in the intervention group (9.5–18.25%). The clinical attachment level (CAL) decreased 1 mm more in the HA therapy group (1–2 mm) than in the no adjunctive treatment group (2–3 mm). The probing depth (PD) was reduced similarly in both groups (3–3.75 mm). Due to the bilateral relationship between diabetes and periodontitis, healthcare professionals seek advancements in managing periodontal inflammation. The results of this study indicate that non-surgical periodontal treatment with HA as an adjunctive agent is worth considering in the therapy for patients with diabetes.

## 1. Introduction

### 1.1. Background

Periodontitis is a complex, multifactorial, chronic inflammatory condition affecting the supportive tissues surrounding the teeth. It is characterized by an imbalance in the oral microbiome and the disruption of tissue equilibrium in susceptible individuals. Clinically, it manifests as inflammation, loss of attachment, irreversible bone degradation and the heightened risk of tooth loss [1,2]. The etiopathogenesis of periodontitis is elucidated by the dysbiotic hypothesis, wherein pathobionts and specific keystone pathogens proliferate relative to symbiotic bacteria and employ immune evasion mechanisms, triggering an inflammatory response and tissue destruction [3].

In recent decades, numerous studies have elucidated the epidemiological links between periodontitis and various non-communicable chronic inflammatory conditions, including cardiovascular, respiratory and metabolic disorders, rheumatoid arthritis, Alzheimer’s disease, type 2 diabetes mellitus (T2DM) and adverse pregnancy outcomes (APOs) [1]. T2DM is characterized by a dysregulated hyperglycemic metabolism and is associated with a decreased lifespan, alongside long-term complications such as cardiovascular disease, diabetic retinopathy and kidney failure. Conversely, periodontitis is recognized as a chronic inflammatory condition with infectious origins. The bidirectional relationship between these diseases has been extensively researched. Individuals with diabetes, which exacerbates glycemic control issues, are more prone to severe periodontitis, and the impact of periodontitis on diabetes may involve bacterial infiltration or its byproducts in systemic circulation, triggering an exaggerated systemic inflammatory response and insulin resistance [2,4,5]. Although simultaneous epidemiological studies on periodontitis and T2DM are lacking, a meta-analysis suggests that individuals with poor glycemic control face twice the risk of developing periodontitis compared to those without it. This underscores the substantial overlap between the two conditions [6].

Recent studies suggest that controlling chronic inflammatory processes induced by periodontitis may mitigate the risk of cardiovascular complications in patients with T2DM. Clinical trials evaluating the effects of conventional periodontal treatment on patients with diabetes have shown promising results, primarily focusing on HbA1c levels as an endpoint measure [7,8]. Effective metabolic control achieved through reductions in glycated hemoglobin, facilitated by antidiabetic medications and lifestyle adjustments, is associated with a decrease in diabetes-related complications, such as myocardial infarction and mortality. Therefore, it is assumed that any reduction in glycated hemoglobin should correspond to a lowered risk of diabetes-associated periodontitis [3,7].

Hyaluronic acid (HA), also known as hyaluronan, stands out as one of the predominant glycosaminoglycans found in the extracellular matrix. In 1934, Karl Meyer and John Palmer isolated HA from the vitreous fluid of a cow’s eye. The term ‘HA’ originates from ‘hyalos’, meaning glass in Greek, referring to its transparent appearance. Structurally, HA comprises two sugar molecules, including uronic acid, forming a linear polysaccharide with repeating disaccharide units. An average healthy adult weighing 70 kg harbors approximately 15 g of HA in the body. With most cells in the human body capable of HA synthesis, its potential in various biological processes becomes evident, making it a promising therapeutic candidate [8]. HA finds utility in the treatment of chronic inflammatory conditions. Although mineralized periodontal tissues contain minimal HA, it is crucial for the extracellular matrix of the gingiva and periodontal ligament. HA accelerates wound healing by influencing the receptors involved in cellular migration, angiogenesis and inflammation regulation. These properties of HA have been harnessed in various periodontal therapies, including non-surgical and surgical interventions, as well as soft and hard tissue regeneration procedures. Additionally, HA plays vital roles in cell signaling, hemostasis, facilitating nutrient and waste exchange between cells, and the extracellular matrix. The potential usefulness of HA in the treatment of periodontitis is connected to its properties and mechanisms of action. It induces wound healing by supporting the growth of fibroblasts, chondrocytes and mesenchymal stem cells and promotes the inflammatory response by inhibiting pro-inflammatory cytokines (IL-1, IL-6 and TNF-α). HA also has the ability to stabilize the granulation tissue matrix, which is important for achieving the periodontal regeneration. It also appears to have anti-angiogenic and immunosuppressive properties, facilitating polymorphonuclear leukocyte access to the wound site, which is essential for dead tissue removal. HA also has some antimicrobial effects and is known for its complete biodegradability [9,10].

According to recent studies, periodontal treatment exerts an influence on metabolic regulation and the mitigation of systemic inflammation in individuals diagnosed with type 2 diabetes. Thus, it may emerge as a novel therapeutic strategy within public health initiatives, aiming to mitigate complications and enhance cardiovascular well-being among patients with T2DM [5,11]. It is emphasized that diabetes management should include the regular assessment and treatment of oral health as an integral aspect of lifelong disease care [7]. Given that periodontitis commonly occurs in patients with diabetes and considering HA is a promising agent in dealing with chronic inflammatory conditions in various tissues, HA adjunctive periodontal therapy in patients with T2DM seems to be worthy of investigation.

### 1.2. Objectives

The aim of this clinical trial was to assess the effectiveness of hyaluronic acid as a local adjunctive in the non-surgical treatment of periodontitis in patients with type 2 diabetes. The clinical parameters of periodontitis as well as the glycated hemoglobin (HbA1) levels were evaluated, and an analysis of the potential differences between the control (placebo) and intervention groups was performed. Our primary outcome was to assess the differences in CAL gain and BOP reduction between the groups. The secondary outcome was to observe the changes in the clinical parameters after both types of periodontal therapy.

### 1.3. Trial Design and Registration

**Figure 1:**
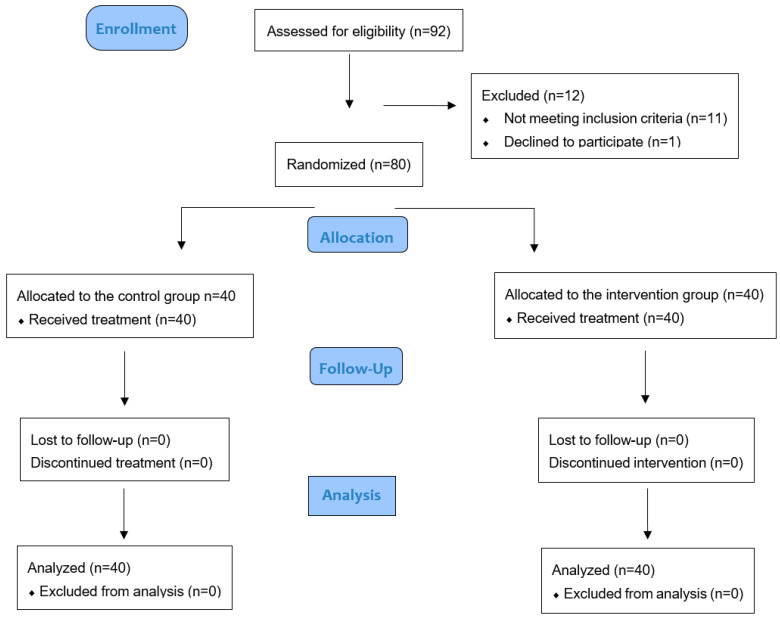
CONSORT 2010 flow diagram.

It was a prospective, single-blinded clinical trial and was conducted at the Department of Periodontal Diseases of the University Dental Clinic in Cracow (Poland). The single operator was calibrated for probing depth (PD) and clinical attachment level (CAL) measurements. A preliminary session of measurements on five patients with periodontitis revealed a correlation coefficient ≥ 0.80. The research was performed in accordance with the Helsinki Declaration, and the participants gave their consent to take part. Approval from the Ethics Committee (Jagiellonian University) was obtained (No. 1072.6120.47.2022). The enrollment took place during regular periodontal appointments. The trial design was based on the CONSORT guidelines (Figure 1). The study protocol is a well-established standard approach in periodontology and was published before the enrollment [12].

### 1.4. Randomization and Blinding

All the participants received code numbers, using randomizing software to allocate them to the control (non-surgical treatment only) or intervention (non-surgical treatment with a local application of hyaluronic acid) groups with an allocation ratio of 1:1. The participants were not informed of the final assignment and remained blinded during the entire study.

## 2. Materials and Methods

### 2.1. Participants

Eighty adult participants with well-controlled T2DM (HbA1c 6–7% (42–53 mmol/mol)) and who were not taking metformin took part in the trial. Before enrollment, patients had completed the first step of periodontal therapy and presented with an approximal plaque index (API) score of less than 25%. Only patients with periodontitis and a minimum two sites with a PD of 4 mm to 5 mm were included. The periodontal diagnosis was based on the Classification of Periodontal Diseases and Conditions (2017) [13]. The exclusion criteria were as follows:

- general diseases other than diabetes;
- antibiotic therapy within the last 6 months;
- non-steroid anti-inflammatory drugs, corticosteroids or multivitamin supplements in the past 3 months;
- smoking in the last 5 years (non-smokers);
- caries;
- epithelial dysplasia or inflammatory lesions of the oral mucosa;
- history of rheumatic disorders, Sjögren disorder, enteritis, asthma or sinusitis;
- pregnancy;
- periodontal treatment within the last 6 months.

### 2.2. Data Collection

The clinical parameters of periodontitis as well as the glycated hemoglobin (HbA1c) levels were recorded at baseline and after 3 months. API, bleeding on probing (BoP), PD and CAL were evaluated at site with a periodontal probe (PCP-UNC 15, Hu-Friedy, Chicago, IL, USA), and the HbA1c data were obtained from current patients’ diabetes records.

### 2.3. Intervention

Both groups were treated according to the full mouth disinfection protocol by the same researcher at one visit. The intervention group underwent standard non-surgical periodontal therapy, followed by HA application to all sites with a PD of 4 mm or more. The control group was treated by standard non-surgical periodontal therapy only (without hyaluronic acid application). Subgingival instrumentation was performed with a Piezon 250 scaler (EMS, Nyon, Switzerland), followed by the use of Gracey curettes (Hu-Friedy, Chicago, IL, USA). None of the patients needed local anesthesia. All the study participants were followed up in line with the standard protocol, and the clinical parameters were evaluated after 12 weeks from baseline. After the trial, consecutive periodontal therapy and/or supportive care were provided as needed.

### 2.4. Hyaluronic Acid Gel

A commercially available product was used in the trial: Hyadent BG^®^ (BioScience GmbH, Dümmer, Germany). It contains a mix of 16 mg/mL cross-linked hyaluronic acid and 2 mg/mL non-cross-linked hyaluronic acid. It is a gel with an average molecular weight of 1 million daltons. The HA in this product comes from bacterial fermentation (*Streptococcus zooepidermicus*), and the cross-linking process is performed at an alkaline pH using BDDE (1,4-Butanediol diglycidyl ether), with a degree of cross linking between 0% and 20%. HA was applied once to the periodontal pockets just after the subgingival instrumentation after the bleeding had stopped using a carpule syringe with a blunt needle (provided by the manufacturer).

### 2.5. Safety Monitoring

The trial participants were followed up in accordance with the standard protocol. During the follow-up, the patients were questioned about any diverse events. After the study, consecutive periodontal supportive care was provided.

### 2.6. Statistical Methods

The minimum required sample size was 46 in total for both groups (level of significance 0.05, power 0.8, the Wilcoxon–Mann–Whitney test). The calculation was based on the primary outcomes [12]: the differences between the two groups after the intervention in terms of the clinical parameters, CAL and BOP. The distributions of the quantitative variables are summarized as the mean, standard deviation, median and quartiles, whereas the distributions of the qualitative variables (sex) are summarized as the number and percentage of occurrence for each of their values. The Chi-squared test (with Yates’ correction for 2 × 2 tables) was used to compare the qualitative variables among the groups. In the case of low values in the contingency tables, Fisher’s exact test was used instead. The Mann–Whitney test was applied to compare the quantitative variables between the two groups. The paired Wilcoxon test was used to compare two repeated measures among the quantitative variables. The significance level for all the statistical tests was set to *p* = 0.05.

## 3. Results

The characteristics of the investigated groups are summarized in Table 1. There were no statistically significant differences between the control and the intervention (study) group at baseline (*p* < 0.05). However, statistically significant differences in some variables were observed between the two groups 12 weeks after the intervention (*p* < 0.05) (Table 2).

**Figure 2:**
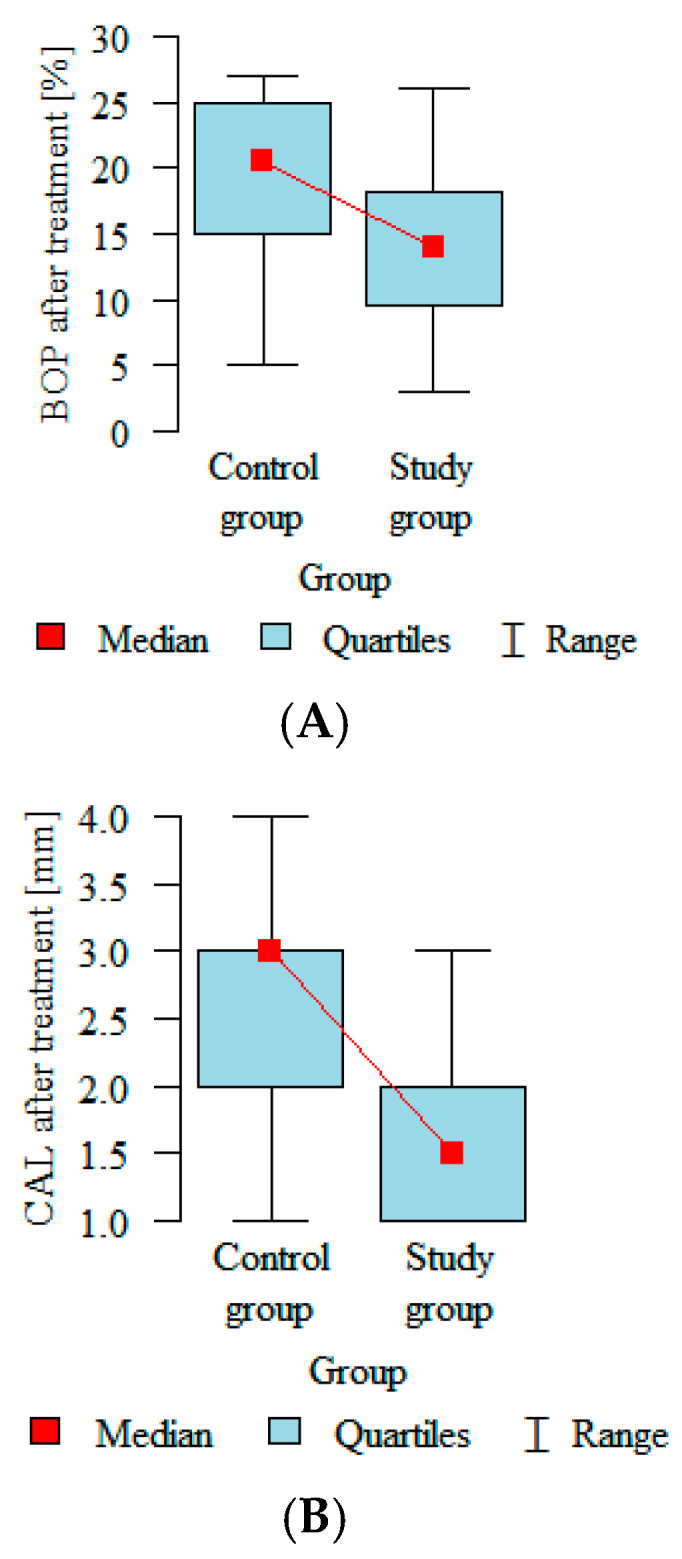
(**A**) BoP and (**B**) CAL in both groups 12 weeks after treatment.

The biggest statistically significant differences between the control and intervention groups after treatment were observed in two variables. Bleeding on probing after 12 weeks from baseline was reduced in both groups but by approximately 5.5% more in the intervention group than in the control group (Figure 2A). A similar pattern was recorded in the clinical attachment level, which decreased 1 mm more in the intervention group than in the placebo group (Figure 2B).

Regardless of the treatment procedure, all the investigated parameters were reduced (*p* < 0.05). The values for the control and study groups are presented in Table 3 and Table 4.

**Figure 3:**
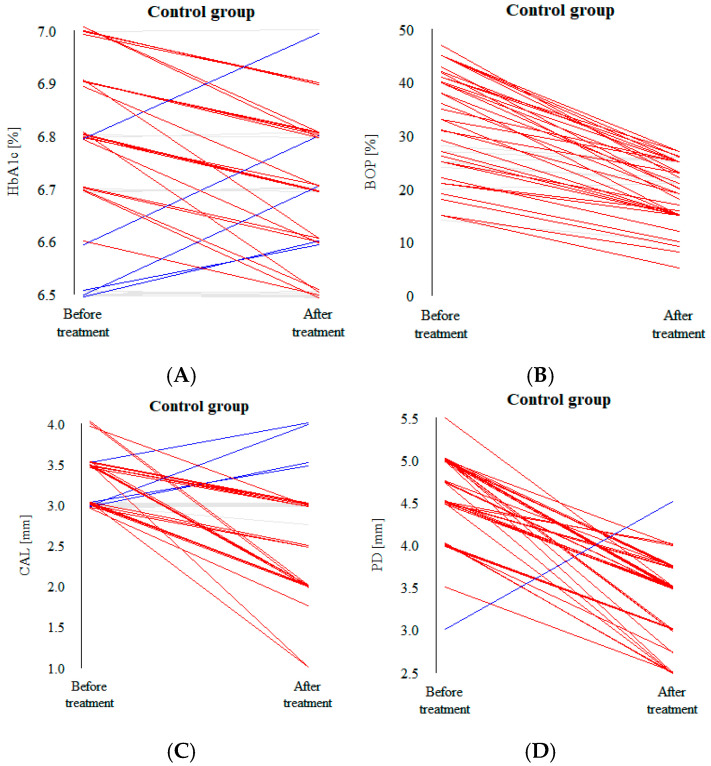
(**A**) HbA1c, (**B**) BoP, (**C**) CAL and (**D**) PD value plots for all the patients in the control group before and after the intervention.

**Figure 4:**
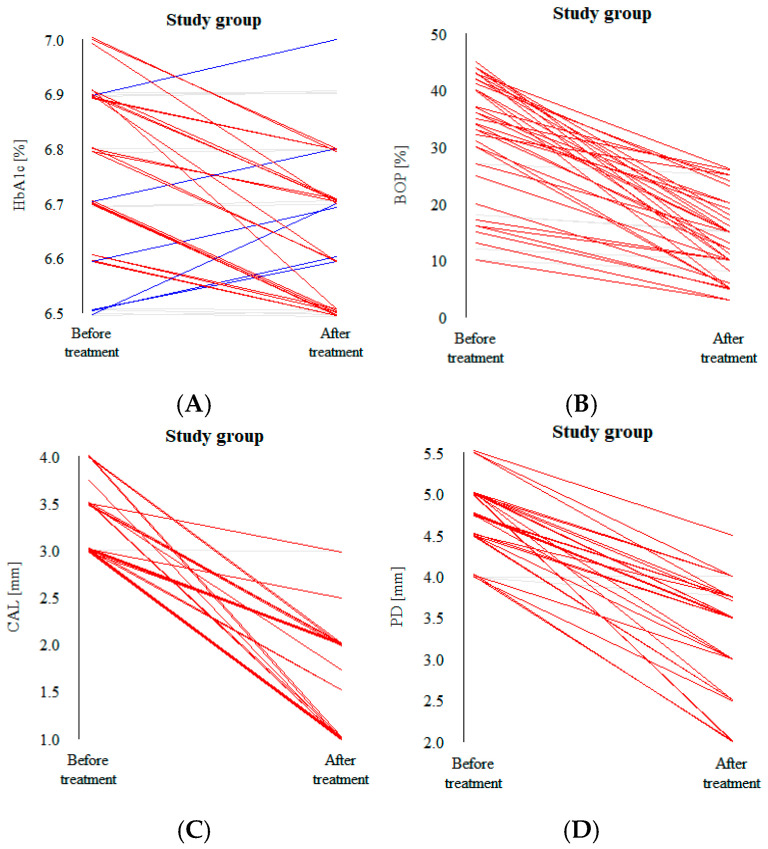
(**A**) HbA1c, (**B**) BoP, (**C**) CAL and (**D**) PD value plots for all the patients in the study group before and after the intervention.

A decrease in all the clinical values after treatment was observed in the vast majority of patients in both groups; this is clearly visible in the parallel coordinate plots (Figure 3A–D and Figure 4A–D). Each line of the plot represents a patient. The red line constitutes a significant reduction, gray a small decrease or no change in value and blue a noticeable increase.

As both groups had a significant decrease in the values of the clinical parameters after the intervention, regardless of the protocol, the differences between those reductions in the control and study categories were evaluated, and they are presented in Table 5 (*p* < 0.05).

**Figure 5:**
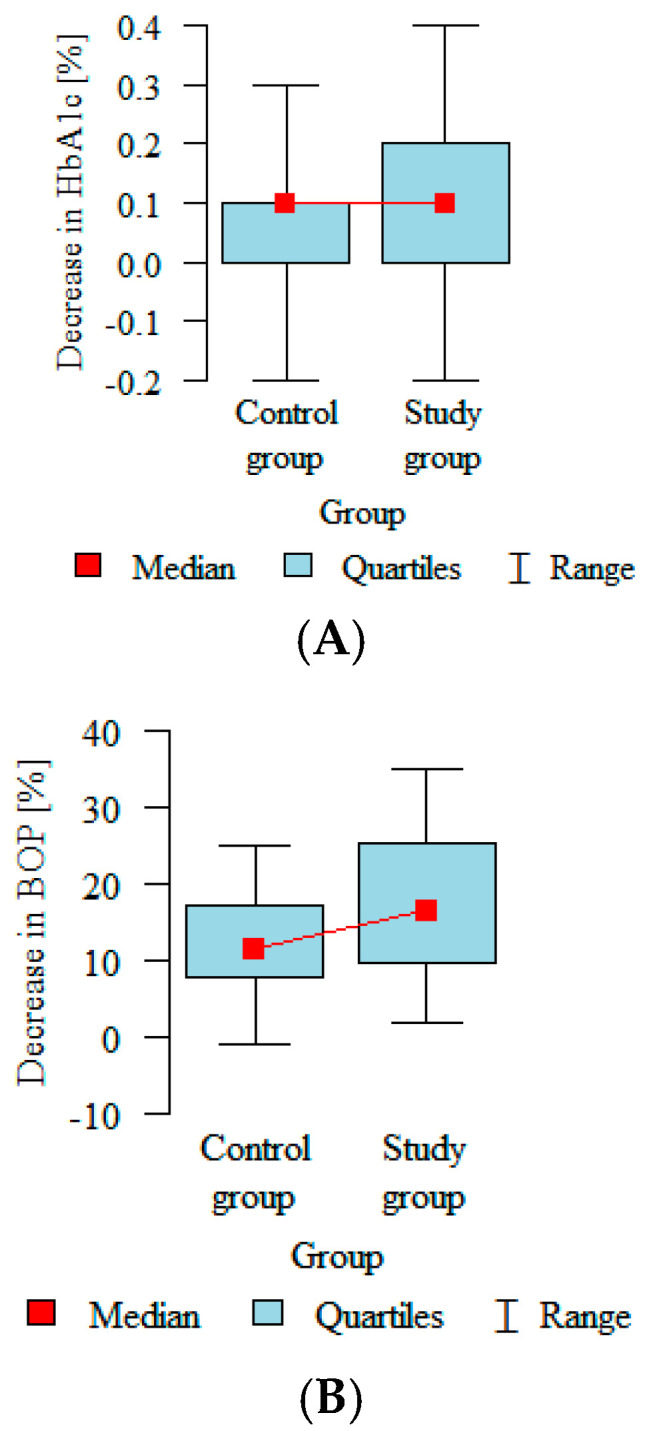

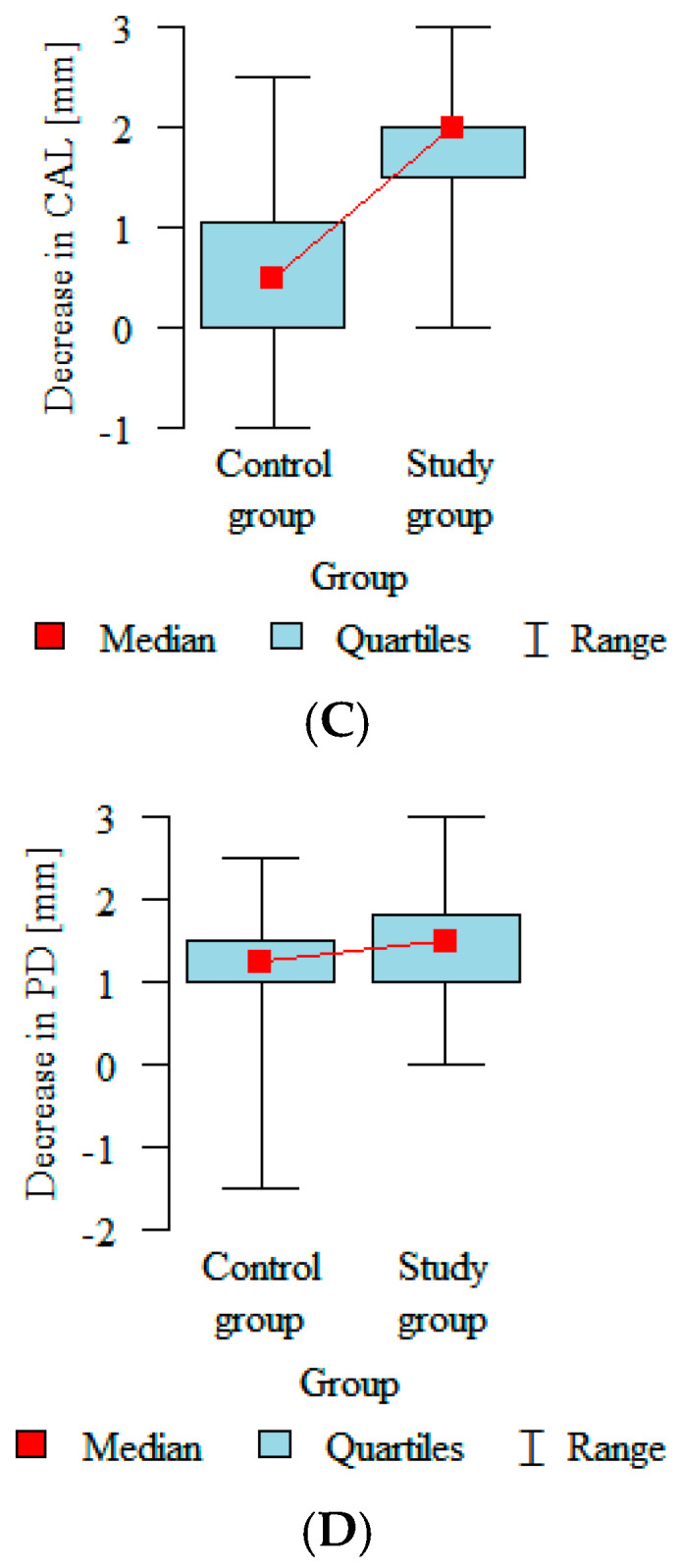
Decrease in (**A**) HbA1c, (**B**) BoP, (**C**) CAL and (**D**) PD values in both groups.

It is noticeable that, after the treatment, there was a significant reduction in all the clinical parameters; however, considering there were no significant differences between the groups before the intervention, the decrease in BOP and CAL in the study group was the highest (Figure 5A–D).

## 4. Discussion

In patients with diabetes, poor glycemic control, defined as glycated hemoglobin (HbA1c) values exceeding 7%, correlates with microvascular and macrovascular complications. Periodontitis is also a common complication for such individuals and is characterized by increased inflammation and tissue destruction. Imbalanced diabetes is attributed to high CRP levels resulting from systemic inflammation caused by AGEs. CRP plays a crucial role in all stages of atherosclerosis, exacerbating endothelial dysfunction and promoting plaque formation and thrombogenesis [2,5,7]. These make patients with diabetes vulnerable to the action of pathogenic microorganisms and changes in the periodontal tissue structure. Elevated CRP levels have also been observed in patients with periodontitis. Studies show that controlling chronic inflammatory processes induced by periodontitis may mitigate the risk of cardiovascular complications in patients with T2DM [8].

HA has emerged as a promising adjunctive treatment in periodontal therapy due to its multipotential mechanism of action [12,14,15,16]. It is important for tissue repair and regeneration, as it enhances fibroblast proliferation and collagen synthesis, promoting healing in periodontal tissues. This is particularly important for patients with diabetes, who often experience delayed healing due to impaired metabolic responses [5,6,12,14,16]. HA also has anti-inflammatory properties, as it can modulate inflammatory processes by inhibiting pro-inflammatory cytokines (IL-1, IL-6, and TNF-α), which may help control the exacerbated inflammatory response seen in periodontitis among patients with diabetes [6,12,14]. Moreover, HA’s ability to retain moisture aids in the maintenance of a healthy environment for oral tissues, which is crucial for effective healing [12,14].

Due to the interconnectedness of diabetes and periodontitis, healthcare professionals on both fronts are motivated to seek advancements in managing periodontal inflammation through improved blood sugar regulation or comprehensive dental interventions to lower glycated hemoglobin levels. There exists evidence indicating that non-surgical periodontal treatment can enhance glycemic control in individuals with diabetes, as evidenced by numerous systematic reviews with meta-analyses, including those by the Cochrane Group in 2015 and umbrella reviews in 2016. These findings collectively suggest that non-surgical periodontal therapy leads to a reduction in glycated hemoglobin levels, typically within 3–4 months post-intervention, ranging from 0.24 to 1.21 percentage points [3,10]. The versatility of HA renders it valuable in periodontal research, spanning a wide range of applications [10]. It has also emerged as a promising adjunctive treatment in periodontal therapy, potentially improving outcomes for patients with diabetes [14,15,16,17].

In our study, there were no statistically significant differences between the control and the intervention groups at baseline in terms of clinical parameters, which made the post-intervention analysis of differences between the variables less complex. Significant distinctions in values were observed between the two groups 12 weeks after the intervention (Table 2). Regardless of the treatment procedure, placebo or HA, all the investigated parameters were reduced (Table 3 and Table 4). Decreases in all the clinical values after treatment were observed in the vast majority of patients in both groups, which is clearly visible in the parallel coordinate plots (Figure 3A–D and Figure 4A–D). Similar results revealing positive outcomes for conservative periodontal therapy in patients with diabetes have been found in many studies. Zanatta et al. [18] investigated the oral microbiota of patients with periodontitis with and without diabetes after non-surgical treatment. The authors’ results suggest a more stable oral microbial community after treatment. The study concluded that there are differences in the structural composition and reaction of the microbiota to therapy, especially between patients with and without diabetes, and a decreased proportion of periodontal pathogens in the oral cavity. Freire et al. [19] investigated an adjunctive non-surgical approach in individuals with diabetes and compared the clinical parameters of patients undergoing adjunctive conservative treatment with those of a control group. The analysis demonstrated that periodontal therapy resulted in a reduction in the probing depth and attachment gain. Another study aimed at analyzing the effect of non-surgical periodontal treatment on salivary enzyme activities, periodontal parameters and glycemic control in individuals with diabetes concluded that there was an improvement in periodontal status [20].

The biggest statistically significant differences in our study between the control and intervention groups after treatment were observed in two variables. The BoP after 12 weeks from baseline was reduced in both groups but by approximately 5.5% more in the intervention group than in the control one (Figure 2). A similar pattern was recorded in the CAL, which decreased 1 mm more in the intervention group than in the placebo group (Figure 3). Many researchers have obtained similar results when evaluating clinical parameters in the course of non-surgical treatment. Pham et al. [21] assessed the effect of periodontal therapy on smokers with diabetes, with a follow-up of 6 months. Individuals from the intervention group significantly improved in all the clinical parameters. Another study evaluated the clinical response to non-surgical treatment in patients with diabetes and observed changes in the mean values of PD, CAL and BoP; the authors noticed improvements in all the parameters [22]. Javed et al. [23] also observed significantly reduced PD, CAL and BoP values in the therapy group compared with baseline.

In our study, both groups had a significant decrease in the clinical parameter values after the intervention regardless of the protocol; the differences between the reductions in the control and study categories were evaluated, and they are presented in Table 5. It is noticeable that, after treatment, there was a significant reduction in all the clinical parameters; however, considering there were no significant differences between the groups before the intervention, the decrease in BoP and CAL in the study group was the highest. The aim of a study by Lin et al. [24] was to assess the differences in periodontitis parameters and inflammatory biomarkers in patients with diabetes after non-surgical periodontal therapy. The authors found significant changes in the PD, BoP, PS and CAL values. Similar results were obtained by Llambés et al., who investigated the response to non-surgical periodontal treatment in individuals with diabetes. They noticed significant improvements in PI, BoP, PD and CAL after therapy [25]. Christgau et al. [26] monitored the clinical, microbiological and immunological effects of conservative periodontal treatment in individuals with diabetes and observed significant positive changes in clinical parameters such as API, PBI, BoP and PD.

There are almost no studies regarding HA adjunctive therapy outcomes in individuals with diabetes; however, this therapy itself is fairly well documented in standard groups of patients, and the results are in line with those in our study [27]. One meta-analysis found mean differences in parameters such as BoP, PD and CAL that were positive for HA adjunctive non-surgical treatment [28]. Another trial investigated a mix of amino acids and sodium hyaluronate gel, which also indicated better outcomes for HA adjunctive therapy compared to a standard treatment protocol [29]. Eick et al. [30] proved a significant PD reduction after HA intervention, and other researchers found a decrease in BoP values [31].

Although in our study there were no statistically significant differences between the control and intervention groups at baseline, and thus, the comparison of the values before and after treatment was not complex, this study still has some limitations. The patients were followed up for 12 weeks only, so we cannot discuss the long-term effects. Moreover, a split-mouth study would probably be a better choice for these parallel groups of patients; however, HA application on the one side of the mouth may lead to its spread through saliva. Additionally, larger-scale clinical trials are needed to validate the results and establish standardized treatment protocols [32,33].

HA seems to be a valuable adjunct in the management of periodontitis in patients with diabetes by improving healing and reducing inflammation. Integrating it into the therapy could lead to significant improvements in periodontal health. Given the shared risk factors and preventive measures between diabetes and periodontitis, future longitudinal studies on HA adjunctive non-surgical periodontal interventions could shed light on their potential to halt the progression of both diseases. Addressing both diabetes and periodontitis through lifestyle modifications could disrupt the bidirectional or cyclic relationship between these conditions, and, as the results of this study suggest, HA adjunctive approach is worth considering, especially in individuals with diabetes [1,2,3,6,8,11,27].

## 5. Conclusions

Regardless of the treatment procedure, all the investigated clinical parameters were reduced after the periodontal therapy. Although there were no statistically significant differences between the groups at baseline, differences in the clinical parameters were observed 12 weeks after the intervention. BOP was reduced by approximately 5.5% more in the intervention group. A similar pattern was recorded for CAL, which decreased 1 mm more in the HA therapy group.

## Figures and Tables

**Table 1:** Characteristics of the investigated groups.

Parameter		Group		*p*
		Control Group (n = 40)	Study Group (n = 40)	
Age [years]	mean ± SD	51.3 ± 8.68	50.98 ± 8.67	*p* = 0.946
	median	51.5	53	
	quartiles	45.5–58.25	46–56.25	
HbA1c before treatment [%]	mean ± SD	6.74 ± 0.18	6.74 ± 0.16	*p* = 0.949
	median	6.8	6.75	
	quartiles	6.57–6.9	6.6–6.9	
BoP before treatment [%]	mean ± SD	31.42 ± 9.86	30.82 ± 11.19	*p* = 0.904
	median	31	33.5	
	quartiles	24.75–40.25	19.5–40.25	
CAL before treatment [mm]	mean ± SD	3.24 ± 0.32	3.36 ± 0.38	*p* = 0.163
	median	3	3.5	
	quartiles	3–3.5	3–3.5	
PD before treatment [mm]	mean ± SD	4.59 ± 0.5	4.74 ± 0.42	*p* = 0.266
	median	4.62	4.75	
	quartiles	4.5–5	4.5–5	
Sex	Female	21 (52.50%)	21 (52.50%)	*p* = 1
	Male	19 (47.50%)	19 (47.50%)	

*p*: Mann–Whitney test for quantitative variables, Chi-squared or Fisher’s exact test for the qualitative variables.

**Table 2:** Clinical parameters in both groups after treatment.

Parameter		Group		*p*
		Control Group (n = 40)	Study Group (n = 40)	
BOP after treatment [%]	mean ± SD	19.27 ± 5.9	13.75 ± 6.91	*p* = 0.001 *
	Median	20.5	14	
	quartiles	15–25	9.5–18.25	
CAL after treatment [mm]	mean ± SD	2.62 ± 0.68	1.56 ± 0.6	*p* < 0.001 *
	Median	3	1.5	
	quartiles	2–3	1–2	
PD after treatment [mm]	mean ± SD	3.33 ± 0.51	3.26 ± 0.65	*p* = 0.795
	Median	3.5	3.5	
	quartiles	3–3.75	3–3.75	
HbA1c after treatment [%]	mean ± SD	6.68 ± 0.15	6.66 ± 0.14	*p* = 0.455
	Median	6.7	6.7	
	quartiles	6.5–6.8	6.5–6.8	

*p*: Mann–Whitney test. * statistically significant (*p* < 0.05).

**Table 3:** Parameter values before and after treatment in general.

Parameter		Before Treatment	After Treatment	*p*
HbA1c [%]	mean ± SD	6.74 ± 0.18	6.68 ± 0.15	*p* = 0.005 *
	median	6.8	6.7	
	quartiles	6.57–6.9	6.5–6.8	
BoP [%]	mean ± SD	31.42 ± 9.86	19.27 ± 5.9	*p* < 0.001 *
	median	31	20.5	
	quartiles	24.75–40.25	15–25	
CAL [mm]	mean ± SD	3.24 ± 0.32	2.62 ± 0.68	*p* < 0.001 *
	median	3	3	
	quartiles	3–3.5	2–3	
PD [mm]	mean ± SD	4.59 ± 0.5	3.33 ± 0.51	*p* < 0.001 *
	median	4.62	3.5	
	quartiles	4.5–5	3–3.75	

*p*: Wilcoxon paired test. * statistically significant (*p* < 0.05).

**Table 4:** Parameter values before and after treatment in the control group.

Parameter		Before Treatment	After Treatment	*p*
HbA1c [%]	mean ± SD	6.74 ± 0.16	6.66 ± 0.14	*p* < 0.001 *
	median	6.75	6.7	
	quartiles	6.6–6.9	6.5–6.8	
BOP [%]	mean ± SD	30.82 ± 11.19	13.75 ± 6.91	*p* < 0.001 *
	median	33.5	14	
	quartiles	19.5–40.25	9.5–18.25	
CAL [mm]	mean ± SD	3.36 ± 0.38	1.56 ± 0.6	*p* < 0.001 *
	median	3.5	1.5	
	quartiles	3–3.5	1–2	
PD [mm]	mean ± SD	4.74 ± 0.42	3.26 ± 0.65	*p* < 0.001 *
	median	4.75	3.5	
	quartiles	4.5–5	3–3.75	

*p*: Wilcoxon paired test. * statistically significant (*p* < 0.05).

**Table 5:** The differences in the parameter value reductions between the control and study groups.

Parameter		Group		*p*
		Control Group (n = 40)	Study Group (n = 40)	
Decrease in HbA1c [%]	mean ± SD	0.06 ± 0.12	0.09 ± 0.13	*p* = 0.463
	Median	0.1	0.1	
	Quartiles	0–0.1	0–0.2	
Decrease in BoP [%]	mean ± SD	12.15 ± 7.11	17.08 ± 9.92	*p* = 0.031 *
	Median	11.5	16.5	
	Quartiles	7.75–17.25	9.75–25.25	
Decrease in CAL [mm]	mean ± SD	0.61 ± 0.8	1.8 ± 0.69	*p* < 0.001 *
	Median	0.5	2	
	Quartiles	0–1.06	1.5–2	
Decrease in PD [mm]	mean ± SD	1.26 ± 0.66	1.49 ± 0.62	*p* = 0.111
	Median	1.25	1.5	
	Quartiles	1–1.5	1–1.81	

*p*: Mann–Whitney test. * statistically significant (*p* < 0.05).

## Data Availability

The raw data supporting the conclusions of this article will be made available by the authors on request.
